# Diisononyl phthalate aggravates allergic dermatitis by activation of NF-kB

**DOI:** 10.18632/oncotarget.13403

**Published:** 2016-11-16

**Authors:** Jun Kang, Jing Song, Shiping Shen, Baizhan Li, Xu Yang, Mingqing Chen

**Affiliations:** ^1^ Hubei Key Laboratory of Genetic Regulation and Integrative Biology, School of Life Sciences, Central China Normal University, Wuhan 430079, Hubei, China; ^2^ Key Laboratory of the Three Gorges Reservoir Region's Eco-Environment, Ministry of Education, Chongqing University, Chongqing 400045, China

**Keywords:** allergic dermatitis, diisononyl phthalate (DINP), NF-kB, oxidative stress, thymic stromal lymphopoietin (TSLP)

## Abstract

Several epidemiological studies have suggested a possible link between exposure to Diisononyl phthalate (DINP) and the development of allergies. These findings remain controversial since there is insufficient scientific evidence to assess the ability of DINP to influence allergic immune responses. In addition, the mechanisms behind DINP-caused allergic diseases have not been fully elucidated. In this study, Balb/c mice were orally exposed to DINP for 3 weeks and were then sensitized with fluorescein isothiocyanate (FITC). We showed that oral exposure to DINP could aggravate allergic-dermatitis-like lesions, indicated by an increase in the number of mast cells, and in increased skin edema in FITC-induced contact hypersensitivity. This deterioration was concomitant with increased total serum immunoglobulin-E and Th2 cytokines. We determined the oxidative damage and the activation of nuclear factor-kb (NF-kB). The data demonstrated that DINP could promote oxidative damage and the activation of NF-kB in the skin. The expression of thymic stromal lymphopoietin and the activation of signal transducer and activator of transcriptions 3, 5 and 6 were enhanced concomitant with exacerbated allergic dermatitis effects and the activation of NF-kB induced by DINP. These effects were alleviated by pyrollidine dithiocarbamate, an inhibitor of NF-kB. The results suggest that oral exposure to DINP aggravated allergic contact dermatitis, which was positively regulated via NF-kB.

## INTRODUCTION

Phthalic acid esters (PAEs) have been widely used as plasticizers. More recently they have been implicated in possibly having a detrimental effect on human health, particularly the endocrine and immune systems [1]. The presence of phthalates in the environment is reported to be associated with asthma (a disease of the respiratory system), and a higher incidence of allergies [2].

Diisononyl phthalate (DINP) is widely used in consumer products as a substitute for other, more toxic plasticizers that are now prohibited in numerous products. It is one of the most-frequently detected particles in multi-surface dust, and in one study of Japanese dwellings, was found in 100% of floor dust samples [3]. Humans are exposed to DINP mainly via dietary intake, and DINP metabolite concentrations can be detected in urine [4].

Compared to dibutyl phthalate (DBP) and di-(2-ethylhexyl) phthalate (DEHP), DINP showed reduced effects on male rat development, and was considered to be an environmentally friendly plasticizer [5]. Several epidemiological studies have, however, suggested an association between exposure to certain phthalate esters (including DINP) and the development of asthma, wheezing, and allergic symptoms [2, 6–8]. Limited evidence supported a link between DINP exposure and atopic dermatitis (AD) [9, 10]. Experimental studies have indicated that several phthalates have an adjuvant effect on basic mechanisms in allergic sensitization [2]. However, the effects of DINP on allergic diseases, and the mechanisms behind these effects have not been fully demonstrated.

An overproduction of T helper type 2 (Th2) mediated cytokines and IgE often result in the development of dermatitis. This imbalance can be caused by excessive sources of oxidative damage induced by the environment, products, microbes, etc [11].

Nuclear factor-κb (NF-κB), as the hub in signal transduction pathways, has extensive biological activities, it participates in inflammation, and immunity, and in cell proliferation and apoptosis of a variety of physiological and pathological processes of gene regulation. NF-κB may play a important role in an organism's response to tissue damage and in the activation of cytokines [12]. Some research has suggested that NF-κB is the molecular culprit that bridges these pathophysiological states and responses [13]. The anti-oxidant pyrollidine dithiocarbamate (PDTC) is a well-known inhibitor of NF-κB [12].

Research has shown that thymic stromal lymphopoietin (TSLP) is derived from epithelial cells, such as keratinocytes, and regulates immunity and inflammation. High expression of TSLP is found in keratinocytes in allergic dermatitis [14, 15]. This cytokine is a key element regulating Th2 responses [16]. TSLP has been shown to promote Th2-type cell responses associated with immunity, and the pathogenesis of many inflammatory diseases, including AD and asthma [17]. It has been shown that environmental factors such as viruses, microbes, parasites, particles from diesel exhaust, and some chemicals trigger TSLP production. Production of TSLP can also be induced or enhanced by Th2-related cytokines, proinflammatory cytokines, and IgE [16]. The upstream of the mouse TSLP transcription initiation site contains two putative NF-κB motifs and is required for inducible TSLP promoter activity [18].

TSLP has been shown to be capable of activating multiple signal transducers and activators of transcription (STATs), such as STAT1, STAT3, STAT4, STAT5, and STAT6 in human dendritic cells (DC) [19]. STAT6 is critical to TSLP maintaining mast cell development, and aggravating mast cell mediated immune responses [20]. STAT5 is required for Th2 allergic responses in both the skin and lungs. Loss of STAT5 in the dendritic cells resulted in the inability to respond to TSLP [21].

FITC is used as a hapten to build the contact hypersensitivity (CHS) model. In this paper we determine the role DINP plays in FITC-induced allergic dermatitis, and investigate the mechanism involved in NF-κB activation.

## RESULTS

### DINP exacerbating allergic dermatitis effects induced by FITC, and PDTC alleviating these effects

To investigate DINP-induced effects, eight groups of mice were gavaged with DINP (2, 20 and 200 mg/(kg·d)or saline for 21 days and then sensitized with or without FITC.

**Figure 1:**
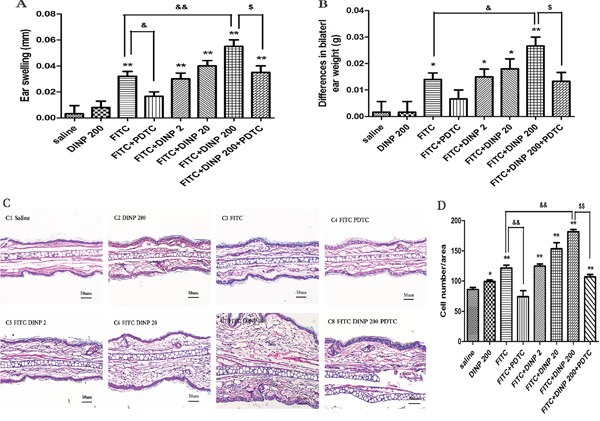
Effects of oral DINP exposure on skin lesions induced by FITC **A.** Thickness of the ears. **B.** Differences in bilateral ear weight. * *p*<0.05, ** *p*<0.01, compared with saline group; && *p*<0.01, compared with FITC group; $$ *p*<0.01, compared with FITC+DINP200 group (n=6). **C.** Histological changes in the ears (stained with H&E). C1, Saline C2, DINP200 C3, FITC C4, FITC+ PDTC C5, FITC+ DINP2 C6, FITC+ DINP20 C7, FITC+DINP200 C8, FITC+DINP200+PDTC. **D.** Evaluated as the number of cells infiltrating. ** p<0.01, compared with saline group; & p<0.05, compared with FITC group; $ p<0.05, compared with FITC+DINP200 group (n=6).

One day after the final challenge we examined ear edemas and compared bilateral ear weights of mice exposed to DINP (Figure 1). Ear edemas and an increase in ear weight were found in the FITC-immunized groups (Figure 1A, 1B). No significant changes were seen in the mice exposed to DINP alone compared with the mice from the saline group. However, exposure to DINP was shown to aggravate ear edema and to significantly increase ear weight when compared with the group exposed to FITC only. This exacerbating effect increased with increasing exposure to DINP (Figure 1A, 1B).

Interestingly, treated with PDTC, a well-known inhibitor of NF-κB, the ear swelling was markedly reduced. This result was seen when the FITC+DINP200+PDTC group was compared with the FITC+DINP200 group (p<0.05) (Figure 1A). Consistent with these findings, bilateral ear weight decreased significantly (p<0.05) when the FITC+DINP-immunized groups were treated with PDTC (Figure 1B).

To further assess histological changes, the right ear from each exposed mouse was stained with hematoxylin and eosin (Figure 1C). Exposure to DINP alone showed no significant pathological changes when compared to the saline group (Figure 1C). The FITC only group showed inflammatory cell infiltration into the skin (Figure 1C, D). Combined administration of DINP and FITC saw an increase in the number of infiltrating inflammatory cells (Figure 1C, 1D). Impressively, when compared with the FITC+DINP200 group, the pathological alterations in the FITC+DINP200+PDTC group are reduced, and fewer inflammatory cells are seen to infiltrate.

These results indicate that exposure to DINP alone does not cause allergic dermatitis, but exposure could exacerbate the allergic dermatitis effects induced by FITC. The effects were alleviated by treatment with PDTC.

### DINP enhanced the total serum IgE

**Figure 2:**
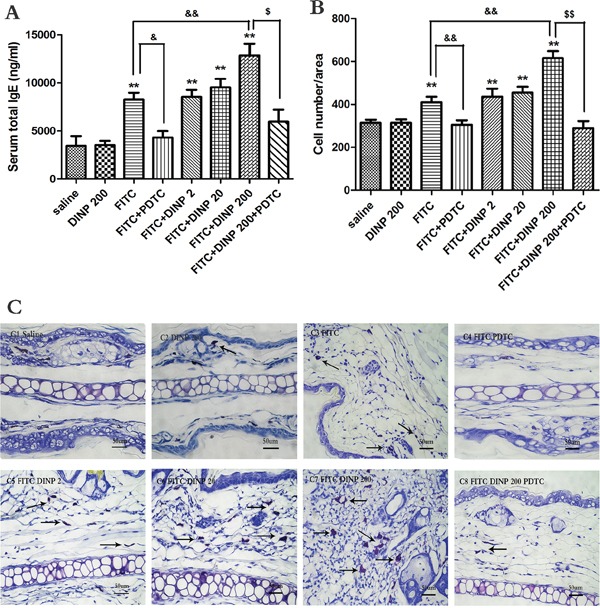
**A.** Total IgE concentrations (ng/ml). **B.** Number of mast cells. **C.** Stained with toluidine blue in the right ear in mice, mast cells were stained purple color (black arrow). C1–C8 and negative represent different exposure groups (Saline, DINP200, FITC, FITC+ PDTC, FITC+ DINP2, FITC+ DINP20, FITC+DINP200, FITC+DINP200+PDTC).

In association with the aggravated allergic dermatitis symptoms, exposure to 200 mg/(kg.d) DINP in combination with FITC markedly exacerbated the total serum IgE, when compared to the FITC sensitized group (Figure 2A). It should be pointed out that exposure to DINP alone (DINP200) did not result in changes in the levels of serum T-IgE when compared to the control group, while the T-IgE levels of the FITC group increased very significantly as well as in the other FITC-immunized groups (FITC+DINP2, FITC+DINP20, FITC+DINP200). Mice treated with PDTC showed a significant decrease in T-IgE levels (p<0.05) (Figure 2A).

**Figure 3:**
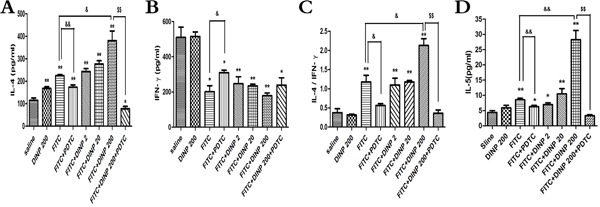
**A.** IL-4 concentrations. **B.** IFN-γ concentrations. **C.** The ratio of IL-4 and IFN-γ. **D.** IL-5 concentrations.* *p*<0.05, ** *p*<0.01, compared with saline group; & *p*<0.05, && *p*<0.01, compared FITC group with FITC+PDTC group and FITC+DINP200 group; $ *P*<0.05, $$ *p*<0.01, compared FITC+DINP200 group with FITC+DINP200+PDTC group (n=6).

As shown in Figure 2B and 2C, more mast cells appeared in the FITC sensitized group, and sensitization was more potent when FITC was administered in conjunction with DINP. The number of mast cells increased when exposed to DINP (Figure 3B and 3C). By comparing the FITC group with the FITC+PDTC group, and the FITC+DINP200 group with the FITC+DINP200+PDTC group, we can see that the mast cell granule levels decreased significantly when the mice were treated with PDTC.

### DINP exacerbated Th2-type contact hypersensitivity

To further determine the inducing mechanism, we measured the levels of the Th1 cytokines (IFN-γ) and the Th2 cytokines (IL-4, IL-5) in the ear homogenate, and from these investigated the ratio of IL-4 to IFN-γ (Figure 3). A significant increase in the IL-4 level and a decrease in the IFN-γ level (Figure 3A, 3B) was observed. This resulted in a marked skew in the ratio (Figure 3C) in the FITC-immunized groups. Also we detected a significant increase in IL-4 levels, and a marked skew in its ratio to IFN-γ when comparing the FITC+DINP200 group with the FITC-only immunized group. Similar to IL-4, the levels of IL-5 were significantly increased when the mice were exposed to DINP and/or FITC, and this enhancement was particularly evident in the FITC+DINP200 group (Figure 3D). These data indicate that DINP exacerbates Th2-type contact hypersensitivity. Mice treated with PDTC exhibited a marked decrease in IL-4 levels and in its ratio to IFN-γ, as can be seen when comparing the FITC group with the FITC+PDTC group, and the FITC+DINP200 group with the FITC+DINP200+PDTC group (Figure 3). A sharp decrease in IL-5 levels was also shown when mice were administered PDTC (Figure 3D).

### DINP exacerbating oxidative stress

**Figure 4:**
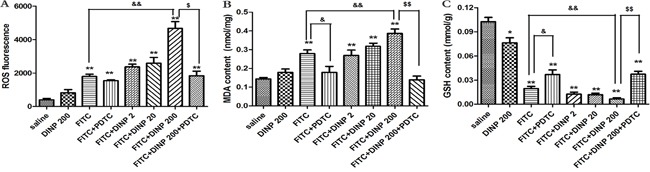
**A.** ROS fluorescence in ear tissue. **B.** MDA concentrations in ear tissue. **C.** GSH concentrations in ear tissue. *p<0.05, **p<0.01, compared with saline group; & *p*<0.05, && *p*<0.01, compared FITC group with FITC+PDTC group and FITC+DINP200 group; $ *P*<0.05, $$ *p*<0.01, compared FITC+DINP200 group with FITC+DINP200+PDTC group (n=6).

The levels of oxidative damage were investigated by measuring ROS, MDA and GSH content in the ear homogenates of exposed animals. As shown in Figures 4A, 4B and 4C, DINP induced a sharp increase in ROS and MDA levels, and a decrease in GSH levels as can be seen by comparing the FITC-immunized groups with the saline group. The DINP induced increase in ROS and MDA levels, and decrease in GSH levels, can be shown by comparing the FITC group with the FITC+DINP200 group (p<0.01). The degree to which ROS and MDA levels increase and GSH levels decrease attenuated by the existence of PDTC is particularly noteworthy. This is shown by comparing the FITC+DINP200 and FITC+DINP200+PDTC groups (p<0.01). Similarly, a significant increase of ROS and MDA and a marked decrease of GSH were found in the spleens of mice immunized with FITC (Supplementary Figure S1). These changes were more obvious when the mice were exposed to DINP in the presence of FITC. Administering PDTC effectively prevented the increase of ROS and the decrease of GSH.

### DINP exacerbating the activation of the NF-κB signal pathway and expression of TSLP

**Figure 5:**
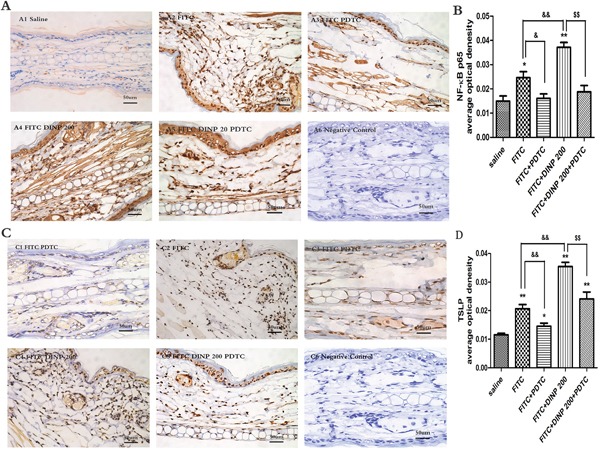
**A.** Immunohistochemistry for NF-κB p65 (phospho S536). **B.** The activation scores of NF-κB p65 (phospho S536). **C.** Immunohistochemistry for TSLP. **D.** The activation scores of TSLP. *p<0.05, **p<0.01, compared with saline group; & *p*<0.05, && *p*<0.01, compared FITC group with FITC+PDTC group and FITC+DINP200 group; $ *P*<0.05, $$ *p*<0.01, compared FITC+DINP200 group with FITC+DINP200+PDTC group (n=6).

We observed a marked activation of NF-κB by investigating phospho-p65 in the FITC-immunized groups (Figure 5A, 5B). Consistent with the aggravated allergic dermatitis symptoms, activation of NF-κB was exacerbated when FITC was applied in combination with DINP. When treated with PDTC, an inhibitor of NF-κB, the activation was inhibited as expected (Figure 5A, 5B).

To certify the regulating role of TSLP, we investigated the expression of TSLP in the ears of mice (Figure 5C, 5D). In association with the activation of NF-κB, expression of TSLP was induced by FITC sensitization in the epidermal cells of ears. An enhancement in TSLP expression was observed when FITC was applied in combination with DINP. The level of TSLP expression was inhibited in the PDTC treated animals (Figure 5C, 5D).

### DINP also exacerbating activation of STAT3, STAT5 and STAT6

**Figure 6:**
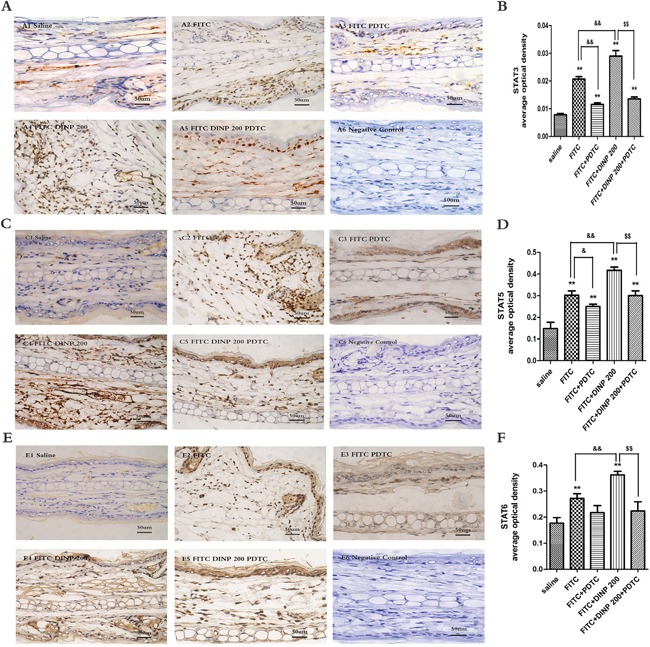
**A.** Immunohistochemistry for STAT3 (phospho Y705). **B.** The activation scores of STAT3 (phospho Y705). **C.** Immunohistochemistry for STAT5 (phospho Y694). **D.** The activation scores of STAT5 (phospho Y694). **E.** Immunohistochemistry for STAT6 (phospho Y641). **F.** The activation scores of STAT6 (phospho Y641). A1–A5, C1–C5, E1–E5 and negative represent different exposure groups (Saline, FITC, FITC+PDTC, FITC+DINP200, FITC+DINP200+PDTC, Negative control). The expression or activation scores were calculated by statistical analysis of optical density of Immunohistochemistry. * *p*<0.05, ** *p*<0.01, compared with saline group; & *p*<0.05, && *p*<0.01, compared FITC group with FITC+PDTC group and FITC+DINP200 group; $ *P*<0.05, $$ *p*<0.01, compared FITC+DINP200 group with FITC+DINP200+PDTC group (n=6).

To further explore the underlying mechanisms associated with DINP, we investigated the activation of STAT3, STAT5 and STAT6 in the ears of mice. As is shown in Figure 6, FITC sensitization induced the activation of STAT3 (Figure 6A, 6B), STAT5 (Figure 6C, 6D), and STAT6 (Figure 6E, 6F) in the epidermal cells of ears. This activation was exacerbated when applied in combination with DINP, and was inhibited when treated with PDTC.

## DISCUSSION

Epidemiological studies have investigated the association between exposure to phthalates and the development of allergic diseases in children. However a lack of objective exposure data limited the interpretation of the epidemiological investigation [22]. The results from acute toxicity studies indicate that DINP produces limited toxicity when applied orally, dermally or via inhalation routes [23]. This finding remains controversial however, since the scientific evidence is insufficient to assess the effect of DINP on immune and allergic responses. This paper shows that oral exposure to 200.0 mg/(kg·day) DINP could aggravate allergic-dermatitis-like lesions related to FITC-induced CHS in mice. This deterioration was concomitant with an increase in total serum IgE and Th2 cytokines such as IL-4. Furthermore, 200.0 mg/(kg·day) DINP potentiated the reproduction of mast cells in the skin in FITC sensitized mice. In addition, DINP could promote oxidative damage in the skin as indicated by the increased production of ROS and MDA, and the decreased production of GSH. These effects were alleviated by the administration of PDTC, an NF-κB inhibitor.

Histopathological changes increased markedly in the FITC sensitized mice exposed to DINP. These features mainly included an increase in basophile infiltration, number of mast cells, and enhanced skin edema, all of which are related to allergic dermatitis in mice.

As expected, activation of NF-κB was inhibited by PDTC treatment. Pretreatment with PDTC also effectively alleviated the development of allergic dermatitis symptoms in FITC sensitized mice exposed to DINP. This successful antagonism also resulted in lower oxidative stress levels. In fact, pretreatment with PDTC was observed to counteract all endpoint effects including high levels of IgE, IL-4, and differences in bilateral ear weight.

The role of oxidative stress in skin allergic disorders has not been fully explored. Decreased blood levels of vitamin E, catalase, and glutathione peroxidase are found in patients with physical urticarias. High levels of ROS are generally detrimental to cells [24, 25]. We observed higher levels of oxidative stress in ear tissue in the ACD model used in this study. When the FITC sensitized mice were co-treated with DINP exposure, the ROS level in ear tissue was further increased.

Depletion of GSH, or production of ROS inhibited the production of the Th1 cytokine and influenced the balance between Th1 and Th2 cells [26]. In this study we show that the intracellular oxidation state was initially induced by FITC sensitization, followed by a disruption of the Th1/Th2 balance, indicated by the production of inflammatory factors IL-4 (typical Th2 cytokine) and IFN-γ (Th1 cytokine). The oxidative state caused by the FITC challenge was aggravated with co-exposure to DINP. With this exacerbating of oxidative damage, there was a pronounced increase in IL-4 levels and its ratio to IFN-γ when FITC was applied in combination with DINP. These results show that DINP promoted Th2 cytokine production in the presence of FITC.

Many situations where ROS is present involve mast cell activation in the allergic inflammatory response. In this study, as ROS levels increased in the FITC-sensitized mice exposed to DINP, we saw an increase in the number of mast cells, as well as an increase in the total serum IgE.

An important target of ROS is NF-κB, which was one of the earliest transcription factors found to be responsive to ROS [27]. We found in this study that increasing exposure to DINP is in line with an increase in the production of oxidative stress and activation of NF-κB. Treatment with PDTC blocked NF-κB activation and also alleviated oxidative damage.

TSLP has been demonstrated to play a critical immunological role in allergic inflammation [14, 28]. It has been shown to play important roles in several Th2-associated disease models, by amplifying and maintaining the Th2 cytokine-mediated immune responses [29–31]. Here we posit that FITC sensitization induced the expression of TSLP in the epidermis and this expression was exacerbated in the presence of DINP.

The production of TSLP can be induced by a high level of activation of the NF-κB pathway [18]. The human TSLP gene promoter contains binding sites for NF-κB. It has been shown that a TSLP deficiency can attenuate allergic reactions by the down-regulation of STAT6 in mast cells [20]. TSLP was shown to activate STAT5, and probably triggers other signaling involved in cell adhesion, cell communication, and lipid metabolism [32]. STAT5 is required for Th2 allergic responses in both the skin and the lungs. Deletion of STAT5 in dendritic cells resulted a loss of response to TSLP [21]. Our results suggest that DINP exacerbated the activation of NF-κB signal pathways, and that is concomitant with the enhanced expression of TSLP and activation of STAT3, STAT5 and STAT6. The enhancement of TSLP expression and activation of the STATs further exacerbates allergic inflammatory responses that include an increase in basophil infiltration; the appearance of more mast cells; and an increase in skin edema. However, the expression of TSLP and the activation of STAT3, STAT5 and STAT6 were inhibited when NF-κB was blocked by PDTC.

**Figure 7:**
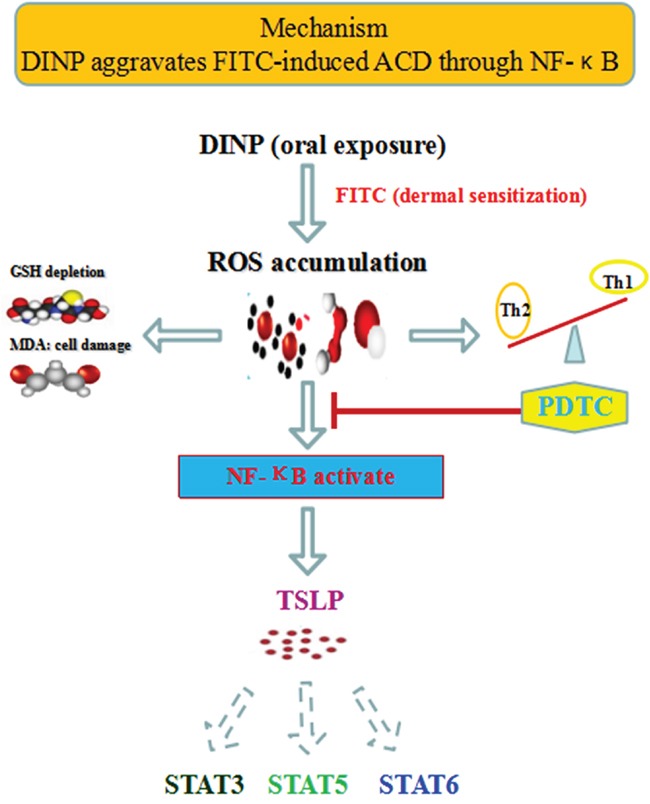
The mechanisms of DINP exacerbating FITC- induced ACD

A possible mechanism to explain these reactions is that DINP, in combination with FITC, triggers the activation of NF-κB by ROS, which in turn promotes the production of TSLP and the activation of STAT3, STAT5 and STAT6, leading to the initiation of the sensitizing process and the exacerbation of allergic dermatitis. Proinflammatory cytokines (Th2-related cytokines such as IL-4, and IgE) contribute to TSLP production and to the activation of the STATs. TSLP production and activation of STATs can be positively regulated via transcription factor NF-κB (Figure 7).

This study demonstrates that long-term oral exposure to DINP aggravates allergic contact dermatitis (ACD) in our mice model. It provides new insights into the onset of allergic dermatitis and the increased prevalence of allergic diseases over the past 30 years. In addition, we found that the activation of NF-κB might play a significant role in allergic inflammation. This finding could help in the quest to devise effective prevention strategies to combat allergic diseases. More studies are needed to understand the molecular mechanisms underlying the aggravation effect caused by DINP so as to prevent these related health problems.

## MATERIALS AND METHODS

### Chemicals

DINP (> 99%), DBP (> 99%), FITC, pentobarbital sodium and formalin solution (4%) were bought from Sigma-Aldrich (St. Louis, MO, USA). Tween-80 and Tween-20 were purchased from Amresco (Solon, OH, USA). Mouse enzyme-linked immunosorbent assay (ELISA) kits for total IgE, IL-4 and (IFN)-γ were obtained from eBioscience (San Diego, CA, USA). MDA and GSH test kits were purchased from Nanjing Jiancheng Bioengineering Institute (Nanjing, China). The protein test kits were provided by Beijing Dingguo Changsheng Biotechnology Co. LTD (Beijing, China).

### Animals

5-6 week old male Balb/c mice were purchased from the Hubei Province Experimental Animal Center (Wuhan. China) and kept in 12 h photoperiod at 23-26 °C and 50-75% humidity. Commercial food and tap water were available ad libitum. Mouse care and experimental procedures were performed under approval from the Office of Scientific Research Management of Central China Normal University, with the certification on the Application for the Animals dated 20 December, 2015 (approval ID: CCNU-IACUC-2016-003).

### Sensitization and challenge

**Figure 8:**
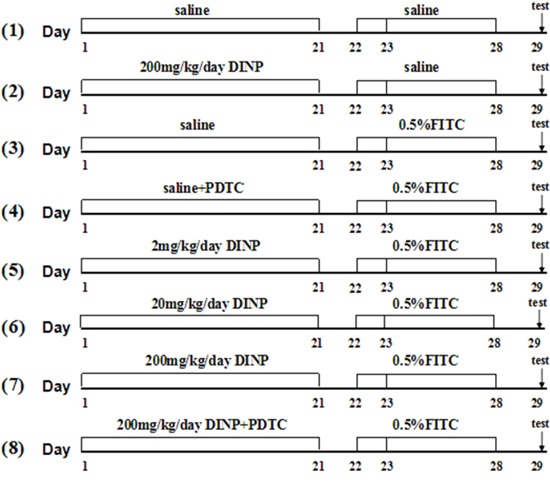
Protocol for exposure and sensitization Male Balb/c mice were gavaged with saline or DINP (2, 20 and 200 mg/(kg·d) from day 1 to 21, then sensitized with 120 ul of saline or 0.5% FITC (in 1:1acetone/DBP) on days 22 and 23, on their shaved backs. On day 28, the mice were challenged with 20 ul of saline or 0.5% FITC to the right ear, and saline or vehicle (1:1 DBP/acetone) to the left ear. Then, baseline ear thickness was measured with vernier calipers. n=6 mice in each group. (1) Saline, (2) DINP200, (3) FITC, (4) FITC+PDTC, (5) FITC+DINP2, (6) FITC+DINP20, (7) FITC+DINP200, (8) FITC+DINP200+PDTC.

48 male Balb/c mice were randomly divided into 8 groups and treated as depicted in Figure 8. In the first stage, Groups (1)-(8) were gavaged with 2, 20, 200 mg/kg bw/day DINP or saline for 21 days, while groups (4) and (8) also were injected with 60 mg/kg bw/day PDTC. Then Mice were sensitized with 100 μl of 0.5% FITC or saline on days 22 and 23 on their shaved backs and challenged with 0.5% FITC on day 28 on their right ears.

### Determination of IgE

On the 29th day, the mice were anesthetized with pentobarbital sodium (100mg/kg, i. P.) by subcutaneous injection within 24 hours after with the last FITC challenge. Serum samples were obtained from heart blood and stored at -80 °C prior to analysis. The total IgE (T-IgE) levels of sera were measured using the IgE ELISA kits. All operations were performed according to the manufacturer's instructions. 15pg / ml was the sensitivity of the kit and each sample was measured in duplicate.

### Ear swelling and weight

Ear thickness was measured using vernier calipers to calculate ear swelling. Ear edema was expressed as (R±L) ± (R0±L0), where R0 and L0 are the thickness of the right and left ear respectively, measured at the beginning of the sensitization day (21d). R and L are the ear thicknesses, measured on day 29. Ear tissue was then punched from the edge of the ear using a mini punch, and weighed to record bilateral ear weight.

### Tissue pathology examination

The right ears of mice were rinsed in ice-cold phosphate-buffered saline (PBS) and fixed for at least 24 hours in 4% formalin at 25 °C. These were then cut into slices, and stained with HE (hematoxylin and eosin), tissue slices were examined using a DM 4000B Microscope (Leica, Berlin, Germany). Image-Pro Plus software (Image-Pro Plus 6.0, Media Cybernetics) was used to analyze the number of inflamed cells in each sample.

### Immunohistochemistry for TSLP, p-STAT3, p-STAT5, p-STAT6, NF-κB p65

The right ears of the mice were collected and fixed overnight in 4% paraformaldehyde. The sections (3μm thickness) were cut and mounted on glass slides. After de-waxing, rehydration and antigen retrieval, the sections were incubated with 0.3% hydrogen peroxide and blocked by appropriate normal serum. Sections were subsequently incubated overnight at 4°C with monoclonal antibodies: anti-phospho-p65 (s536) (1:200, Abcam, MA, USA), anti-TSLP (1:200, GeneTex, CA, USA), anti-phospho-STAT3 (Y705) (1:100, Abcam, MA, USA), anti-phospho-STAT5 (Y694) (1:100, Abcam, MA, USA), and anti-phospho-STAT6 (Y641) (1:100, Abcam, MA, USA) respectively. Then antibody binding was detected by biotinylated immunoglobulins and avidin-biotin peroxidase complex. The reaction product was visualized by DAB complexes. The negative control was obtained by omitting the primary antibody. Sections were washed again, counterstained with hematoxylin, dehydrated, cleared, and mounted in DPX (Sigma-Aldrich).

### Oxidative stress biomarker measurement

The ear tissue was homogenized in 10 ml/g of PBS and centrifuged at 10,000rpm for 10 min. The supernatant was collected to determine the levels of ROS, MDA and GSH. The specific methods and procedures used have been previously described [33].

### ELISA

Collected 24 h after the final exposure, a 10% tissue homogenate was produced by using 10 ml/g of ice-cold PBS at pH 7.5 with homogenized ear tissues in a glass homogenizer on ice. After centrifuging at 10,000 rpm for 10 min at 4 °C, the supernatant was collected to assess the levels of IL-4 and IFN-γ using commercial ELISA kits according to the manufacturer's instructions. The sensitivities of the kits were 15 pg/ml for both IL-4 and IFN-γ. Concentrations were tested in duplicate for each sample.

### Statistical analysis

Data are presented as mean ± SEM. Statistical graphs were generated by GraphPad Prism 5.0. The significance of variation among groups was determined by one-way ANOVA combined with Tukey's multiple comparison tests. A p-value <0.05 is regarded as statistically significant and p-value <0.01 is regarded as extremely significant.

## SUPPLEMENTARY MATERIALS FIGURES AND TABLES



## References

[1] Salapasidou M, Samara C, Voutsa D (2011). Endocrine disrupting compounds in the atmosphere of the urban area of Thessaloniki, Greece. Atmos Environ.

[2] Bornehag CG, Nanberg E (2010). Phthalate exposure and asthma in children. Int J Androl.

[3] Ait Bamai Y, Araki A, Kawai T, Tsuboi T, Saito I, Yoshioka E, Kanazawa A, Tajima S, Shi C, Tamakoshi A, Kishi R (2014). Associations of phthalate concentrations in floor dust and multi-surface dust with the interior materials in Japanese dwellings. Sci Total Environ.

[4] Koch HM, Lorber M, Christensen KL, Palmke C, Koslitz S, Bruning T (2013). Identifying sources of phthalate exposure with human biomonitoring: results of a 48h fasting study with urine collection and personal activity patterns. Int J Hyg Environ Health.

[5] Boberg J, Christiansen S, Axelstad M, Kledal TS, Vinggaard AM, Dalgaard M, Nellemann C, Hass U (2011). Reproductive and behavioral effects of diisononyl phthalate (DINP) in perinatally exposed rats. Reproductive Toxicology.

[6] Wang IJ, Lin CC, Lin YJ, Hsieh WS, Chen PC (2014). Early life phthalate exposure and atopic disorders in children: a prospective birth cohort study. Environ Int.

[7] Ait Bamai Y, Shibata E, Saito I, Araki A, Kanazawa A, Morimoto K, Nakayama K, Tanaka M, Takigawa T, Yoshimura T, Chikara H, Saijo Y, Kishi R (2014). Exposure to house dust phthalates in relation to asthma and allergies in both children and adults. Science of The Total Environment.

[8] Smit LAM, Lenters V, Høyer BB, Lindh CH, Pedersen HS, Liermontova I, Jönsson BAG, Piersma AH, Bonde JP, Toft G, Vermeulen R, Heederik D (2015). Prenatal exposure to environmental chemical contaminants and asthma and eczema in school-age children. Allergy.

[9] Koike E, Yanagisawa R, Sadakane K, Inoue K, Ichinose T, Takano H (2010). Effects of diisononyl phthalate on atopic dermatitis in vivo and immunologic responses in vitro. Environ Health Perspect.

[10] Wu Z, Li J, Ma P, Li B, Yang X (2016). Long-term dermal exposure to diisononyl phthalate exacerbates atopic dermatitis through oxidative stress in an FITC-induced mouse model. Frontiers in Biology.

[11] Schwartz JR, Henry JP, Kerr KM, Mizoguchi H, Li L (2015). The role of oxidative damage in poor scalp health: ramifications to causality and associated hair growth. Int J Cosmetic Sci.

[12] Satriano J, Schlondorff D (1994). Activation and attenuation of transcription factor NF-kB in mouse glomerular mesangial cells in response to tumor necrosis factor-alpha, immunoglobulin G, and adenosine 3’:5’-cyclic monophosphate. Evidence for involvement of reactive oxygen species. J Clin Invest.

[13] Tak PP, Firestein GS (2001). NF-kappaB: a key role in inflammatory diseases. J Clin Invest.

[14] Leyva-Castillo JM, Hener P, Jiang H, Li M (2013). TSLP produced by keratinocytes promotes allergen sensitization through skin and thereby triggers atopic march in mice. J Invest Dermatol.

[15] Nakajima S, Igyarto BZ, Honda T, Egawa G, Otsuka A, Hara-Chikuma M, Watanabe N, Ziegler SF, Tomura M, Inaba K, Miyachi Y, Kaplan DH, Kabashima K (2012). Langerhans cells are critical in epicutaneous sensitization with protein antigen via thymic stromal lymphopoietin receptor signaling. J Allergy Clin Immunol.

[16] Takai T (2012). TSLP expression: cellular sources, triggers, and regulatory mechanisms. Allergol Int.

[17] Ziegler SF, Artis D (2010). Sensing the outside world: TSLP regulates barrier immunity. Nat Immunol.

[18] Lee HC, Ziegler SF (2007). Inducible expression of the proallergic cytokine thymic stromal lymphopoietin in airway epithelial cells is controlled by NFkappaB. Proc Natl Acad Sci U S A.

[19] Arima K, Watanabe N, Hanabuchi S, Chang M, Sun SC, Liu YJ (2010). Distinct signal codes generate dendritic cell functional plasticity. Sci Signal.

[20] Han NR, Oh HA, Nam SY, Moon PD, Kim DW, Kim HM, Jeong HJ (2014). TSLP induces mast cell development and aggravates allergic reactions through the activation of MDM2 and STAT6. J Invest Dermatol.

[21] Bell BD, Kitajima M, Larson RP, Stoklasek TA, Dang K, Sakamoto K, Wagner KU, Kaplan DH, Reizis B, Hennighausen L, Ziegler SF (2013). The transcription factor STAT5 is critical in dendritic cells for the development of TH2 but not TH1 responses. Nat Immunol.

[22] Kimber I, Dearman RJ (2010). An assessment of the ability of phthalates to influence immune and allergic responses. Toxicology.

[23] Lithner D, Nordensvan I, Dave G (2012). Comparative acute toxicity of leachates from plastic products made of polypropylene, polyethylene, PVC, acrylonitrile-butadiene-styrene, and epoxy to Daphnia magna. Environ Sci Pollut Res Int.

[24] Bowler RP, Crapo JD (2002). Oxidative stress in allergic respiratory diseases. J Allergy Clin Immunol.

[25] Westerink RH (2014). Modulation of cell viability, oxidative stress, calcium homeostasis, and voltage- and ligand-gated ion channels as common mechanisms of action of (mixtures of) non-dioxin-like polychlorinated biphenyls and polybrominated diphenyl ethers. Environ Sci Pollut Res Int.

[26] You HH, Chen SH, Mao L, Li B, Yuan Y, Li R, Yang X (2014). The adjuvant effect induced by di-(2-ethylhexyl) phthalate (DEHP) is mediated through oxidative stress in a mouse model of asthma. Food and Chemical Toxicology.

[27] Schieber M, Chandel NS (2014). ROS function in redox signaling and oxidative stress. Curr Biol.

[28] Leyva-Castillo JM, Li M (2014). Thymic stromal lymphopoietin and atopic diseases. Revue Française d’Allergologie.

[29] Larson RP, Zimmerli SC, Comeau MR, Itano A, Omori M, Iseki M, Hauser C, Ziegler SF (2010). Dibutyl phthalate-induced thymic stromal lymphopoietin is required for Th2 contact hypersensitivity responses. J Immunol.

[30] Moniaga CS, Jeong SK, Egawa G, Nakajima S, Hara-Chikuma M, Jeon JE, Lee SH, Hibino T, Miyachi Y, Kabashima K (2013). Protease activity enhances production of thymic stromal lymphopoietin and basophil accumulation in flaky tail mice. Am J Pathol.

[31] Siracusa MC, Saenz SA, Hill DA, Kim BS, Headley MB, Doering TA, Wherry EJ, Jessup HK, Siegel LA, Kambayashi T, Dudek EC, Kubo M, Cianferoni A (2011). TSLP promotes interleukin-3-independent basophil haematopoiesis and type 2 inflammation. Nature.

[32] Voehringer D (2012). Basophil modulation by cytokine instruction. Eur J Immunol.

[33] Yan B, Li J, Guo J, Ma P, Wu Z, Ling Z, Guo H, Hiroshi Y, Yanagi U, Yang X, Zhu S, Chen M (2016). The toxic effects of indoor atmospheric fine particulate matter collected from allergic and non-allergic families in Wuhan on mouse peritoneal macrophages. Journal of Applied Toxicology.

