# Supplementary material for: Diisononyl phthalate aggravates allergic dermatitis by activation of NF-kB
Source: Oncotarget. 2016 Nov 16;7(51):85472–82. doi: 10.18632/oncotarget.13403 (PMC5356750; doi:10.18632/oncotarget.13403)
Supplement: Supplementary file 1 [file oncotarget-07-85472-s001.pdf]

Diisononyl phthalate aggravates allergic dermatitis by activation of NF-κB

SUPPLEMENTARY FIGURE

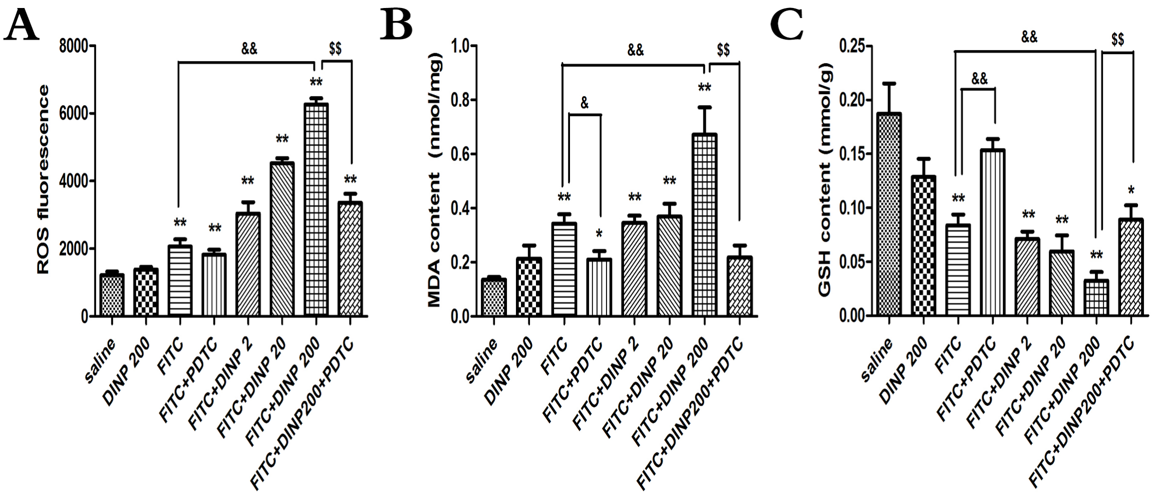

Supplementary Figure S1: DINP exacerbating oxidative stress in spleen. A. ROS fluorescence in spleen. **B.** MDA concentrations in spleen. **C.** GSH concentrations in spleen. * $p<0.05$, ** $p<0.01$, compared with saline group; & $p<0.05$, && $p<0.01$, compared FITC group with FITC+PDTC group and FITC+DINP 200 group; $ $P<0.05$, $$ $p<0.01$, compared FITC+DINP 200 group with FITC+DINP 200+PDTC group (n=6).
